# DNA damage in blood lymphocytes in patients after ^177^Lu peptide receptor radionuclide therapy

**DOI:** 10.1007/s00259-015-3083-9

**Published:** 2015-06-06

**Authors:** Uta Eberlein, Carina Nowak, Christina Bluemel, Andreas Konrad Buck, Rudolf Alexander Werner, Harry Scherthan, Michael Lassmann

**Affiliations:** Department of Nuclear Medicine, University of Würzburg, Oberdürrbacher Str. 6, 97080 Würzburg, Germany; Bundeswehr Institute of Radiobiology affiliated to the University of Ulm, 80937 Munich, Germany

**Keywords:** γ-H2AX, 53BP1, Biological dosimetry, Peptide receptor radionuclide therapy, Absorbed dose to blood, DSB focus assay, DNA damage, ^177^Lu, Ionizing radiation

## Abstract

**Purpose:**

The aim of the study was to investigate DNA double strand break (DSB) formation and its correlation with the absorbed dose to the blood lymphocytes of patients undergoing their first peptide receptor radionuclide therapy (PRRT) with ^177^Lu-labelled DOTATATE/DOTATOC.

**Methods:**

The study group comprised 16 patients receiving their first PRRT. At least six peripheral blood samples were obtained before, and between 0.5 h and 48 h after radionuclide administration. From the time–activity curves of the blood and the whole body, residence times for blood self-irradiation and whole-body irradiation were determined. Peripheral blood lymphocytes were isolated, fixed with ethanol and subjected to immunofluorescence staining for colocalizing γ-H2AX/53BP1 DSB-marking foci. The average number of DSB foci per cell per patient sample was determined as a function of the absorbed dose to the blood and compared with an in vitro calibration curve established in our laboratory with ^131^I and ^177^Lu.

**Results:**

The average number of radiation-induced foci (RIF) per cell increased over the first 5 h after radionuclide administration and decreased thereafter. A linear fit from 0 to 5 h as a function of the absorbed dose to the blood agreed with our in vitro calibration curve. At later time-points the number of RIF decreased, indicating progression of DNA repair.

**Conclusion:**

Measurements of RIF and the absorbed dose to the blood after systemic administration of ^177^Lu may be used to obtain data on the individual dose–response relationships in vivo. Individual patient data were characterized by a linear dose-dependent increase and an exponential decay function describing repair.

**Electronic supplementary material:**

The online version of this article (doi:10.1007/s00259-015-3083-9) contains supplementary material, which is available to authorized users.

## Introduction

Patients who develop neuroendocrine and other tumour entities overexpressing peptide receptors can be treated with peptide receptor radionuclide therapy (PRRT) leading to longer survival and improved quality of life [1]. This molecular targeted radiation therapy involves the systemic administration of a radiolabelled peptide designed to target the overexpressed receptors on tumour cells with high affinity and specificity [1]. In the past 15 years, PRRT with the radiolabelled somatostatin receptor agonists ^90^Y-DOTA-D-Phe-Tyr3-octreotide (^90^Y-DOTATOC), ^177^Lu-DOTA-D-Phe-Tyr3-octreotate (^177^Lu-DOTATATE) and ^177^Lu-DOTA-D-Phe-Tyr3-octreotide (^177^Lu-DOTATOC) have been successfully used to target metastatic and inoperable neuroendocrine tumours (NET) expressing somatostatin receptor subtype 2 [1–3]. PRRT has also been used to treat meningioma [4–7] and thyroid cancer [8–10].

The haematological toxicity of this treatment is an issue, since it has been observed particularly after the administration of ^90^Y-labelled DOTA compounds [11], whereas treatment with ^177^Lu-labelled compounds delivers lower absorbed doses to the bone marrow [12]. In agreement with this, patients receiving ^177^Lu-DOTATATE/DOTATOC did not show haematological toxicity with the exception of one with grade 3 leukopenia and thrombocytopenia [13]. In any case, systemic administration of therapeutic radionuclide activities will lead to DNA damage because of the protracted whole-body irradiation.

PRRT also provides the opportunity to study the effects of prolonged systemic β- and γ-irradiation after administration of ^177^Lu in vivo. In this setting, all organs including the blood are irradiated by β-particles emitted from circulating ^177^Lu and from penetrating γ-radiation originating from activity dispersed throughout the body. The absorbed dose and dose rate to the blood after systemic administration of ^177^Lu-labelled compounds is assessed by defining the time–activity curves in the blood and the whole body, integrating the corresponding time–activity curves and calculating the absorbed dose [14].

Furthermore, radiation exposure can be correlated with biological effects, including DNA damage that is assessed biodosimetrically in terms of chromosome aberrations or DNA double strand breaks (DSBs). DSBs are critical cellular lesions that can result from genotoxins such as ionizing radiation or chemical compounds [15–17]. Their formation in nuclear chromatin results in rapid phosphorylation of the histone H2 variant H2AX, called γ-H2AX [15, 18, 19]. DSBs also recruit the damage sensor 53BP1 to the chromatin surrounding the DSBs [20–24], which leads to 53BP1 and γ-H2AX colocalization in the chromatin surrounding a DSB [20, 22, 25–27]. Therefore, radiation-induced DSBs can be addressed by microscopically visible DNA damage protein foci that display both γ-H2AX and 53BP1 immunofluorescence [27]. With progression of DSB repair, γ-H2AX and 53BP1 foci disappear [28]. The immunofluorescence staining for colocalizing γ-H2AX/ 53BP1 foci and their quantification as DSBs has been termed the DNA damage focus assay [26, 27].

At present, there are only two studies that have quantified radiation-induced DNA damage focus formation after treatment of differentiated thyroid cancer (DTC) with the isotope ^131^I, either using radiation-induced colocalizing γ-H2AX and 53BP1 foci [29] or γ-H2AX foci only [30]. A more recent study has addressed γ-H2AX foci formation after ^177^Lu therapy of NET [31]. In these studies elevated levels of radiation-induced DNA damage foci were observed after treatment, but a dose–response relationship could not be established.

The aims of the present study were therefore, (a) to establish a methodology for describing absorbed doses to the blood in patients after PRRT similar to the formalism developed for radioiodine therapy of thyroid cancer [32], (b) to describe the temporal and dose-dependent behaviour of the DNA damage focus assay in radiation treatment-naive patients after their first PRRT with ^177^Lu, (c) to compare the in vivo dose response in the first hours after therapy with an in vitro calibration curve established recently in our laboratory [27], and (d) to describe and quantify the decay of foci at later time-points after administration of the radiopharmaceutical as a potential measure of the repair of radiation-induced double stranded DNA damage in vivo.

## Materials and methods

### Research design and subjects

Patients referred to our centre for initial treatment with ^177^Lu-labelled DOTATATE/DOTATOC were included in this study. The treatment was done on a compassionate use basis in patients with an advanced stage of their disease or who were without any other therapeutic options. Haematological disease was an exclusion criterion. During the week prior to treatment with radiolabelled peptides patients were requested not to receive ionizing radiation for diagnosis and/or treatment.

Before PRRT, patients were hospitalized in our ward to check their medical condition. On the treatment day, a mixture of l-arginine and l-lysine was given immediately before PRRT over 3.5 to 4 h to reduce the dose to the kidneys [1]. The ^177^Lu-labelled DOTATATE/TOC activity was then delivered using a perfusion system over a period of 20 min. The end of the administration process was chosen as the starting point of the study. After the start of treatment the patients stayed in our ward for 2 days.

### Blood sampling and activity determination in blood samples

Blood samples were drawn in all patients prior to administration and nominally at 1, 2, 3, 4, 24 and 48 h after administration using Li-heparin blood collecting tubes (S-Monovette, Sarstedt, Germany). For exact quantification of the blood activity concentration, an aliquot of 0.1 mL of each heparinized blood sample was measured in a well counter (Canberra, Germany) or in a high-purity germanium detector (Canberra, Germany). The counting efficiencies of the detectors were determined by repeated measurements of a NIST-traceable standard. The measured values were decay-corrected to the time of blood sampling.

### Blood sample preparation for the DNA damage focus assay

Separation and fixation of white blood cells and counting of the foci followed the protocol described by Eberlein et al. [27] the foci being stained according to the procedure described by Lassmann et al. [29] and Lamkowski et al. [26]. The average numbers of radiation-induced damage foci (RIF) per cell were obtained by subtracting the background number of foci for each counted sample. A detailed description of this method is provided in the Supplementary material.

### Measurement of the whole-body retention

Whole-body activity retention was determined in all patients by combining external dose rate measurements and whole-body gamma camera scans. For the first measurement, the patients were asked not to micturate or defaecate after administration of the radiopharmaceutical. Prior to subsequent measurements they were asked to micturate. The dose-rate measurements were performed using a ceiling-mounted shielded survey meter (automess–Automation und Messtechnik, Germany) at a fixed distance of 2.5 m above the patient’s bed. The patient measurements were carried out immediately after administration and at least two times per day thereafter. The data were normalized to the first initial measurement. In addition, whole-body scans with a gamma camera (Symbia T2; Siemens Healthcare, Germany) were performed 1 h and 24 h after administration (medium energy collimator, energy window 208 keV ±10 %). In selected patients, an additional scan was performed 4 h after treatment. The gamma camera whole-body retention was calculated by normalizing the geometric mean of subsequent background-corrected anterior and posterior counts to the initial measurement. Both datasets were combined to obtain the whole-body retention curve for each patient.

### Calculation of the time-integrated activity coefficients and the absorbed doses

A biexponential fit function was adequate to determine the function describing the activity as a function of time for the whole body and blood. The time-integrated activity coefficients for the whole body and activity concentration in blood (τ_total body_(*t*) and τ_ml of blood_(*t*)) were calculated by integrating the respective time–activity functions over time. Note the unit of τ_total body_ is hours and the unit of τ_ml of blood_ is hours per millilitre because we considered only a small blood volume (0.1 ml) for our measurements in the well counter. The absorbed doses were calculated using a procedure analogous to the EANM standard operational procedure for dosimetry in the treatment of DTC [32] using the following equation:

1 $$ {\overline{\mathit{\mathsf{D}}}}_{\mathsf{blood}}\ \left(\mathit{\mathsf{t}}\right)={\mathit{\mathsf{A}}}_{\mathsf{0}}\cdot \left(\frac{\mathsf{85.3}\ \mathsf{Gy}\cdot \mathsf{ml}}{\mathsf{GBq}\cdot \mathsf{h}}\cdot {\tau}_{\mathsf{ml}\ \mathsf{of}\ \mathsf{blood}}\ \left(\mathit{\mathsf{t}}\right)+\frac{0.00185}{\mathit{\mathsf{w}}{\mathit{\mathsf{t}}}^{\raisebox{1ex}{$2$}\!\left/ \!\raisebox{-1ex}{$3$}\right.}}\kern0.5em \frac{\mathsf{Gy}\cdot {\mathsf{kg}}^{\raisebox{1ex}{$2$}\!\left/ \!\raisebox{-1ex}{$3$}\right.}}{\mathsf{GBq}\cdot \mathsf{h}}\cdot {\tau}_{\mathsf{t}\mathsf{otal}\ \mathsf{body}}\left(\mathit{\mathsf{t}}\right)\right) $$

where *A*_0_ is the administered activity and *wt* is the patient’s weight in kilograms. The method is described in more detail in the Supplementary material.

### Modelling the time-dependency of focus induction and disappearance

Most in vitro and in vivo studies of ionizing radiation-induced DSB formation have indicated a linear relationship between the number of microscopically visible RIF and the absorbed dose [15, 18, 27, 33], the dose–length product in CT examinations [34, 35] or the total body dose in radiotherapy [36]. For our set-up and staining procedure we also observed a linear dose–response relationship between the absorbed dose to the blood and the number of RIF per cell in an in vitro experiment [27].

As has been pointed out by Dale and Fowler [37], sublethal DNA damage repairs monoexponentially, assuming that the rate of repair at any instant is directly proportional to the number of unrepaired lesions remaining (first-order process). However, the same authors found that monoexponential repair could not completely explain the observations made in several clinical studies [37]. The easiest way to account for this would be to introduce a multiexponential model with different repair rates. Another model introduced by Fowler [38] and by Dale et al. [39] assumes that the rate of repair of damaged lesions is proportional to the square of their number (second-order process); a finding, however, that has not been confirmed yet for the DNA damage focus assay. Studies of the DNA damage focus assay by Horn et al. [40] and Mariotti et al. [41] have revealed that the number of RIF per cell decreases over time with the onset of DNA repair, following a biexponential model [40, 41]. Hence, we decided to describe the decrease in the number of RIF per cell over time with a biexponential model. Therefore, the time dependency of the number of RIF per cell as a function of the time-dependent absorbed dose and the disappearance of foci can be described in the low absorbed dose range by a linear dose-dependent increase using the input of our in vitro calibration curve and biexponential decay representing DNA repair:

2 $$ N(t)=\left(a+m\cdot b\cdot \overline{D}{}_{\mathrm{blood}}(t)\right)\cdot \left(k\cdot {e}^{-\lambda \cdot t}+\left(1-k\right)\cdot {e}^{-\upsilon \cdot t}\right) $$

where:

- *N*(*t*) is the number of foci at time *t*.
- *m* is an adjustable parameter to account for the variability in patient dosimetry with respect to the in vitro calibration established by Eberlein et al. [27].
- *a* and *b* are constants describing the in vitro calibration curve [27] representing the number of RIF per cell as a function of the mean time-dependent absorbed dose $\overline{D}$_blood_(*t*) (*a* = 0.0363 RIF/cell; *b* = 0.00147 RIF/cell · mGy^-1^).
- $\overline{D}$_blood_(*t*) is the mean absorbed dose to the blood (Eq. 1).
- *λ* and *υ* are patient-specific adjustable parameters describing the decay rate of foci.
- *k* is an adjustable parameter describing the fraction of damage assigned to different repair rates. Since we performed only two measurements at time-points >12 h, for this study, we set *k* = 1.

### Statistics

Origin (version 9.1G + 2015G, Origin Lab Corporation) was used for data analysis and statistical evaluation. The normal distribution of the datasets was tested using the Shapiro-Wilk test. RIF per cell value data sets at different time-points were compared using the paired *t* test. Differences were considered significant for *p* < 0.05.

## Results

### Patients

Of 18 patients enrolled in the study, 16 were included (mean age 61.2 ± 10.5 years, Table 1). The patient demographics are given in Table 1. Ten of the patients presented with NET. Other diseases included adrenocortical cancer, meningioma and papillary thyroid carcinoma. All patients except one presented with metastases. Thirteen patients were referred to our centre because of progressive disease. The pretreatment of these patients included surgery, chemotherapy, radiotherapy, sandostatin, mitotane and everolimus. All pretreatment was discontinued >1.5 months before PRRT.

**Table 1:** Patient demographic data

Patient ID	Age (years)	Weight (kg)	Activity (GBq)	Compound	Tumour	Local recurrence	Location of metastases	Indication	Pretreatment	Grade/Ki67 (%)
Lu1	65.8	96	7.03	DOTATOC	NET, jejunum	No	Lymph nodes	PD	Surgery	G1/2
Lu2	56.5	94	6.41	DOTATOC	NET, pancreatic	No	Liver, bone	PD	CTx, everolimus	G2
Lu3	72.8	92	7.94	DOTATOC	Papillary thyroid cancer	No	Lymph nodes, lung	PD	Surgery, RIT, CTx	–
Lu4	67.9	82	6.60	DOTATOC	Unknown primary	No	Bone, liver	PD	Sandostatin	G2
Lu5	54.3	100	6.90	DOTATOC	Adrenocortical cancer	Yes	Lymph nodes, peritoneal	PD	Surgery, CTx	–
Lu6	67.1	94	7.60	DOTATOC	NET, lung	No	Lymph nodes, pleura, bone	PD	CTx	>90 %
Lu7	56.4	41	7.00	DOTATOC	Adrenocortical cancer	Yes	Lung, peritoneal	PD	Surgery, CTx, mitotane	–
Lu8	30.6	95	7.30	DOTATOC	NET, lung	No	Bone, liver, lymph nodes	PD	CTx, RTx	–
Lu10	68.5	82	7.05	DOTATOC	NET, ileum	No	Liver, lung, lymph nodes	PD	Sandostatin	–
Lu11	67.2	70	7.13	DOTATOC	NET, ileum	No	Liver, lymph nodes	PD	Surgery	–
Lu12	61.5	70	7.70	DOTATATE	Meningioma	Yes	–	PD	RTx	–
Lu13	61.4	64	7.70	DOTATATE	NET, pancreatic	No	Lymph nodes	PT	–	G2
Lu14	48.8	60	7.54	DOTATATE	NET, lung	No	Lymph nodes	PT	–	100 %
Lu16	67.5	79	7.01	DOTATATE	Unknown primary	No	Liver	PT	–	x
Lu17	61.3	75	6.97	DOTATATE	NET, ileum	No	Lymph nodes, liver, peritoneal	PD	Surgery, sandostatin	G2
Lu18	72.2	67	6.99	DOTATATE	NET, gastric	No	Liver	PD	Sandostatin	G2

*PD* progressive disease, *PT* primary treatment, *NET* neuroendocrine tumour, *CTx* chemotherapy, *RIT* radioiodine therapy, *RTx* radiotherapy

For PRRT the patients received ^177^Lu-labelled DOTATATE (patients 1 – 11) or DOTATOC (patients 12 – 18) intravenously (mean activity 7.2 ± 0.4 GBq) via a perfusion system over 20 min. Patients were then admitted to our ward and discharged 2 days later. All blood samples and measurements were taken during this time period. All patients responded well to the treatment or showed disease stabilization at follow-up and showed no therapy-related or study-related adverse effects.

### DNA damage foci

**Fig. 1:**
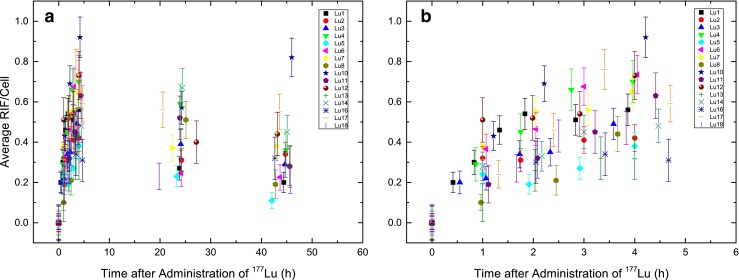
Average RIF per cell as a function of time after administration of ^177^Lu: **a** all data, time-points 0 – 48 h; **b** detailed view of time-points 0 – 5 h

Peripheral blood lymphocytes were immunofluorescently stained for γ-H2AX/53BP1 DNA damage foci and manually counted for colocalizing DSB-marking foci. The average numbers of RIF per cell were calculated subtracting the number of background foci for the different patient samples (Fig. 1) as a function of time after administration of the radiopharmaceutical. The average number of RIF per cell increased in the first hours after therapy, decreasing at later time-points (Fig. 1, Table 2). The actual time-points differed slightly because of variations in management of individual patients. The mean numbers of RIF per cell were 0.55 at 4 h, 0.42 at 24 h, and 0.43 at 48 h after administration of ^177^Lu. Distribution analysis of the numbers of RIF per cell identified the 48-h value in patient Lu10 as an outlier that was excluded from further analysis. All three datasets were distributed according to a Gaussian distribution (Shapiro-Wilk test). Applying the paired *t* test to all time-points revealed that there were statistically significant differences among the numbers of RIF per cell at these different time-points (*p* < 0.008) confirming the observation that the number of RIF per cell decreased 5 h after administration of the radiopharmaceutical.

**Table 2:** Number of RIF per cell and absorbed doses at different time-points

Patient ID	Background foci values	Nominal time-points						
		4 h		24 h		48 h		
		Absorbed dose (mGy)	No. of RIF per cell	Absorbed dose (mGy)	No. of RIF per cell	Absorbed dose (mGy)	Gamma contribution (%)	No. of RIF per cell
Lu1	0.03	27.1	0.56	44.4	0.27	53.6	19	0.20
Lu2	0.01	23.7	0.42	50.7	0.31	68.0	29	0.34
Lu3	0.06	42.0	0.49	63.6	0.39	76.8	16	0.29
Lu4	0.20	16.4	0.70	40.5	0.59	57.4	33	0.36
Lu5	0.02	45.1	0.38	85.2	0.23	110.4	11	0.11
Lu6	0.04	29.1	0.74	55.2	0.25	75.5	18	0.23
Lu7	0.03	58.9	0.65	86.4	0.37	95.5	7	0.38
Lu8	0.16	8.4	0.44	34.1	0.51	57.5	34	0.19
Lu10	0.05	36.5	0.92	65.7	0.57	85.0	18	0.82^a^
Lu11	0.33	39.3	0.63	67.7	0.52	80.2	16	0.28
Lu12	0.35	43.0	0.73	65.8	0.40	73.3	10	0.44
Lu13	0.40	36.3	0.32	64.9	0.36	77.3	15	0.28
Lu14	0.11	54.0	0.48	81.0	0.67	88.2	8	0.45
Lu16	0.39	40.7	0.31	76.6	-	84.7	9	0.32
Lu17	0.11	33.3	0.59	59.9	0.56	67.1	12	0.55
Lu18	0.10	22.0	0.47	68.6	0.23	106.5	22	0.21
Mean	0.15	34.7	0.55*	63.1	0.42*	78.6	17	0.34*
Standard deviation	0.14	13.3	0.17	15.4	0.15	16.4	9	0.17

*p < 0.008

^a^Outlier

### Dosimetry

**Fig. 2:**
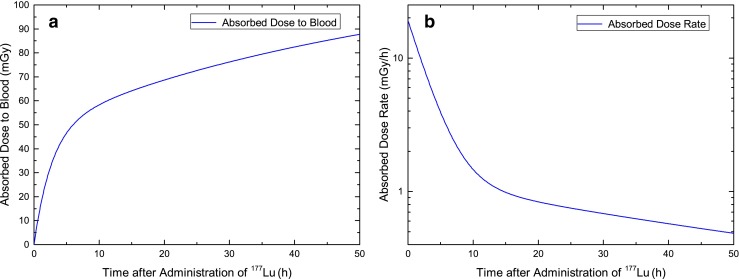
Absorbed dose to the blood (**a**) and corresponding dose rate (**b**) in a selected patient (Lu3)

**Fig. 3:**
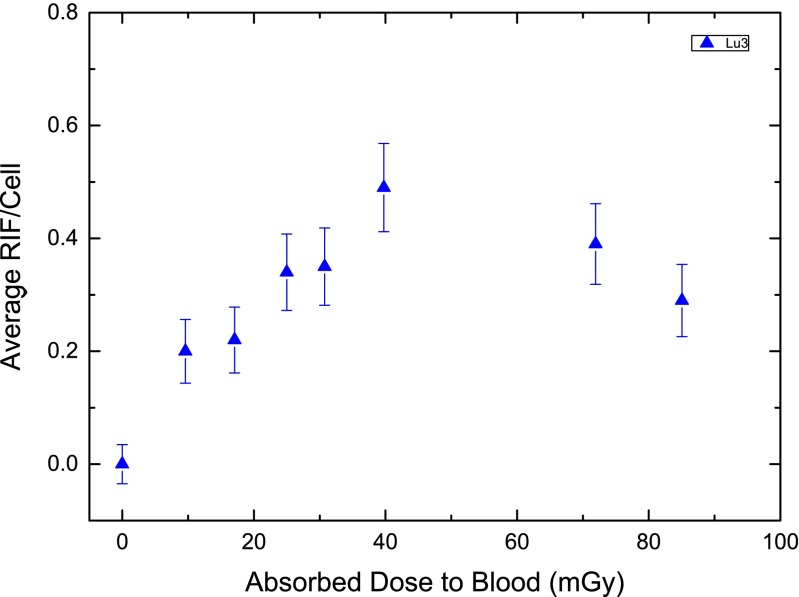
Average RIF per cell as a function of the absorbed dose to the blood in patient Lu3. The blood sampling times were: 0, 0.6, 1.1, 1.7, 2.3, 3.6, 24.1 and 44.7 h

The absorbed doses to the blood in the individual patients were calculated according to Eq. 1. The mean absorbed dose was 34 ± 13 mGy at 4 h, 63 ± 15 mGy at 24 h and 79 ± 16 mGy at 48 h after administration (Table 2). The relative contribution of the penetrating radiation to the absorbed dose 48 h after treatment was less than 34 % in all patients. In the first few hours after treatment the contribution was below 10 % (data not shown). Due to the lack of data at time points > 2 days, and the associated uncertainty in extrapolating the data to time infinity, the total absorbed dose was not calculated. The absorbed dose to the blood increased steeply in the first few hours after therapy (Fig. 2). In most patients, 50 % of the absorbed dose to the blood at 48 h was reached within the first 5 h. In accordance with this observation, the dose-rate decreased until it was less than 0.5 mGy/h after 48 h. For comparison, Fig. 3 shows the average number of RIF per cell for this example patient (Lu3) as a function of the absorbed dose to the blood.

### In vivo calibration of the DNA damage focus assay

**Fig. 4:**
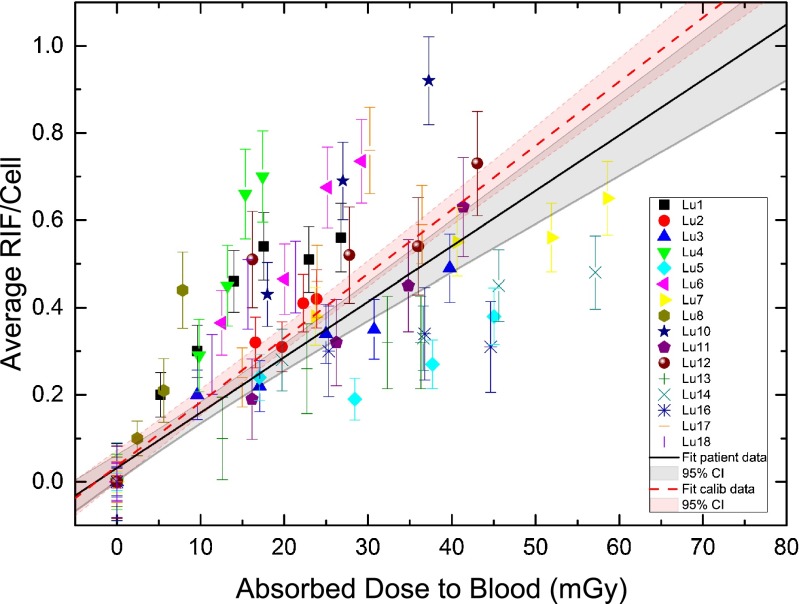
Average number of RIF per cell as a function of the absorbed dose to the blood (over the first 5 h after ^177^Lu administration). The *black line* is the linear fit to our ^177^Lu patient data (in vivo calibration curve) including the 95 % confidence interval (CI), and the *red line* is the in vitro calibration curve taken from Eberlein et al. [27] including the 95 % CI

The average numbers of RIF per cell as a function of the absorbed dose in each patient for the first 5 h after treatment are shown in Fig. 4. The first datasets up to 5 h after administration of the radiopharmaceutical were pooled. A linear fit to our ^177^Lu patient data (in vivo calibration) resulted in: *y* = 0.0321 + 0.0127·*x*, where *y* denotes the number of RIF per cell and *x* the absorbed dose to the blood in milligray (*R*^2^ = 0.72). The standard error of the *y*-axis intercept was ± 0.0152 RIF per cell and the standard error of the slope was ± 0.0009 RIF per cell · mGy^−1^. The *y*-axis intercept value in this case takes the standard deviation of the background (maximum value ± 0.09 RIF per cell) value into account. Therefore, we did not force it to zero, although, no RIF per cell would be expected at 0 mGy. For absorbed doses above 10 mGy the influence of the *y*-axis intercept on the number of RIF per cell was less than 20 %. The resulting in vivo calibration curve for our ^177^Lu patient data including the 95 % confidence interval is also shown in Fig. 4. In a previous study we obtained an in vitro calibration curve for ^131^I and ^177^Lu from blood samples of volunteers [27]. For comparison this in vitro calibration curve is shown in Fig. 4. The relative deviation of the slopes between the in vitro calibration curve and the in vivo data is 14 %.

### Modelling

Individual fits of the patient data were performed according to Eq. 2 using the datasets for the biokinetics of blood (*λ*_*bl* 1,2_, *A*_*bl* 1,2_) and the total body (*λ*_*tb* 1,2_ and *A*_*tb*1,2_) provided in Table S1 as well as the values of the in vitro calibration [27] (*a* and *b*). Variable parameters to be fitted were *m* (adjustable parameter to account for the variability in the patient dosimetry with respect to the in vitro calibration) and the repair rate *λ* (Table S1). For this study we chose *k* = 1 in Eq. 2, thus describing the repair in terms of a monoexponential function only. The reason was that we obtained only two blood samples per patient at 24 h and 48 h after administration, which was not sufficient for an adequate approximation of a biexponential function. Because of the lack of late data points only a monoexponential fit was possible; therefore, we could not provide a second repair rate *ν*.

**Fig. 5:**
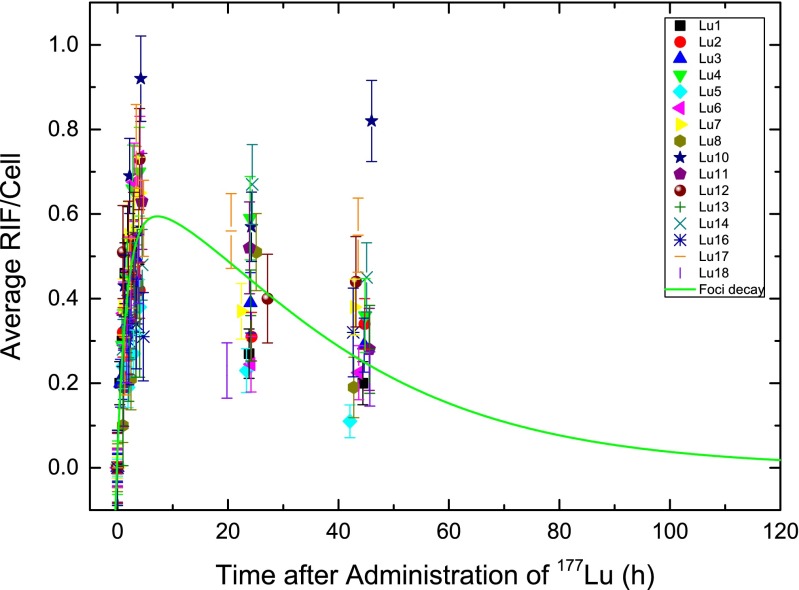
Average number of RIF per cell as a function of time after ^177^Lu administration, including the modelled decay function using the mean values of all foci–decay fits according to Eq. 2. Parameters are the mean values presented in Table S1

In general, the data followed the in vitro calibration curve for the first 5 h after treatment. The mean value of the fitted parameter *m* which accounts for the variability in patient dosimetry with respect to the in vitro calibration was 1.28 ± 0.66 (minimum 0.55, maximum 2.84). The mean decay rate (*λ*) in all patients was 0.0379 ± 0.0187 h^−1^ (minimum 0.014 h^−1^, maximum 0.084 h^−1^) corresponding to 18.3 h effective decay time. The maximum of this fitted curve including the average parameters of all patients was at 7.2 h after administration of ^177^Lu. After 75.5 h the average number of RIF per cell dropped below the maximum standard deviation of the baseline value of 0 ± 0.09 RIF per cell (Fig. 5). The standard deviation of the background foci included the counting error. Therefore, each data point was considered with appropriate error propagation, even the baseline value of RIF per cell at 0 mGy.

## Discussion

This study provides a first clear correlation between the average number of RIF per cell and the absorbed dose to the blood after PRRT up to 5 h after administration of the β-emitter ^177^Lu. Absorbed doses to the blood of nearly all patients were less than 100 mGy 48 h after treatment. Concomitant generation of a significantly elevated number of radiation-induced DSB foci was noted up to 48 h after therapy (end of our follow-up).

Recently, Denoyer et al. [31] analysed the kinetics of γ-H2AX foci formation in peripheral blood lymphocytes of 11 patients undergoing PRRT. The number of γ-H2AX foci in 50 – 100 cells per sample was determined before and up to 72 h after treatment using a dedicated computational algorithm (TGIR foci-counting software) [42]. Denoyer et al. observed a variable response among patients, but, unlike our findings, detected no clear relationship between the absorbed dose to the blood and the number of γ-H2AX foci. The correlations between γH2AX foci yield and the absorbed doses presented by the authors are poor or, in the case of bone marrow, lead to a negative number of γ-H2Ax foci for bone marrow doses <100 mGy, a fact which is neither explained nor discussed [42].

While there are many studies showing elevated foci levels after exposure to ionizing radiation [33, 40, 43–48], only a limited number of studies have shown the effects of the therapeutic or diagnostic use of radionuclides [29–31, 35]. For internal irradiation in molecular radiotherapy the time-course of the induction and the number of RIF are different from those following external irradiation, since after radionuclide administration cells are irradiated not only for seconds or minutes, but are continuously irradiated over a longer period of time with a permanently changing dose rate [29].

Blood-based dosimetry in PRRT using ^177^Lu shows several differences from blood-based dosimetry of radioiodine as, for example, described by Hänscheid et al. [49]. The absorbed dose to the blood from the penetrating radiation of ^177^Lu is almost an order magnitude lower than that from ^131^I. Therefore, the contribution of penetrating radiation to the absorbed dose to the blood is in most patients less than 20 % at 48 h after administration of ^177^Lu, and even less in the first few hours after administration (data not shown). However, it is unknown how the absorbed dose to the blood from penetrating radiation changes due to uptake in the liver, spleen, tumour and metastases. The mean observed absorbed dose to the blood after 48 h in the ^177^Lu-treated patients in this study was lower than the values reported by Lassmann et al. [29] for ^131^I-treated thyroid carcinoma patients. In agreement with Sandström et al. [50], this can mainly be attributed to the fact that the activity in the blood has a shorter half-life in the first hours after ^177^Lu administration than after ^131^I administration. For clinical reasons, the blood sampling times, particularly for the first samples, were variable and therefore, the first phase of the blood time–activity curve might not have been a satisfying representation in all patients, a fact which may have affected the correlation with the RIF induced in the first few hours after administration of the radiopharmaceutical.

Overall, the absorbed doses to the blood were low in all patients receiving ^177^Lu therapy, indicating that the likelihood of haematological toxicity is rather low for this treatment, in agreement with the findings of Sandström et al. in 200 patients [50]. In a previous radioiodine DTC study we also observed the decay in the average number of RIF per cell after radioiodine therapy of DTC [29], with the highest number of RIF per cell being observed 2 h after therapy; however, no blood samples were available for the next few hours, while the decrease of in the number of RIF per cell [29] at later time-points mirrored the findings of the present study. Doai et al. [30] observed no temporal dependency of γ-H2AX foci and the absorbed dose to the blood, probably because their first blood collection was at 96 h after radioiodine administration, a time period that is, according to the current results and our experience, too late to reveal a direct dose–response relationship. May et al. [35] investigated the induction of γ-H2AX foci by a PET tracer (^18^F-FDG) used in nuclear medicine diagnostics. However, in that study the FDG PET (β-exposure) was followed by exposure to X-rays from CT. Thus, the exposure to X-rays has likely obscured any RIF induced by the radiopharmaceutical.

The observed numbers of RIF per cell for the first hours after treatment obtained in this work are in good agreement with the in vitro calibration curve [27] developed in our laboratory. The slight differences in slope in relation to the in vitro calibration curve can be explained by the results of two recent studies by Hänscheid et al. [51, 52] in which the authors investigated the absorbed doses to the blood from compounds that do not bind to the blood. According to these results [51] the gamma component is underestimated for ^177^Lu by a factor of about 2 as compared to the model we assumed. In addition, when a realistic distribution of vessel sizes is taken into account this results in a beta absorbed dose that is lower than the maximum energy deposited by beta particles [52]. A specific model for the case of PRRT describing the absorbed dose to the blood is so far not available.

We are aware that the numbers of RIF per cell strongly depend on the background values of the patients. As can be seen in Table 2, these values underlie strong intrinsic variations, possibly related to age, lifestyle, nutrition, genetics background and stress. Cell fixation as well as staining artefacts can also play a role in variability [48]; however, we controlled for this by including internal 0 Gy and 1 Gy controls in all staining reactions. Only staining reactions in which control values were similar were evaluated. Other studies have also shown a high variability in background values [29, 31, 46, 53, 54]. Overall, these findings show that the DNA damage focus assay may be used as an in vivo dosimeter in the first hours after incorporation of beta-emitting radioisotopes.

The disappearance of RIF as a function of time has been quantitatively investigated by Horn et al. [40] and by Mariotti et al. [41], with the former describing the disappearance using a biexponential decay function with a short decay rate of 0.350 h^−1^ (77 %) and a longer-lived component of 0.018 h^−1^ (23 %). Mariotti et al. also described, for a single acute absorbed dose of 1 Gy, biexponential decay with a short component (relative contribution 91 %, decay constant 0.23 h^−1^) and a second phase that showed almost no decay (relative contribution 9 %, 3.32 × 10^−12^ h^−1^). In the current study, for logistic reasons, we were only able to include two blood samples per patient obtained >5 h after administration of ^177^Lu. Hence, we decided to describe the decay using a monoexponential function only. Our RIF per cell decay rates lie, with one exception (patient Lu14: 0.014 h^−1^), within the range of values reported by Horn et al. [40]. The value for patient Lu14 could potentially be interpreted as showing a lower repair rate of DNA damage than in the other patients; however, this finding could also be explained by the variability in the individual patient dosimetry data. In patients with a very low repair rate (patients Lu14 and Lu16) or a very high repair rate (patients Lu6 and Lu18), no obvious link between these findings the condition of the patients, and the pretreatments or stage of disease could be found.

Further studies with more patients and different tracer kinetics in the blood are needed to better identify patients with deviating repair rates.

### Conclusion

This study shows the effect of ionizing radiation on blood lymphocytes after systemic administration of a radiopharmaceutical in the course of PRRT as a function of the absorbed dose to the blood. For the first time a clear correlation between the average number of RIF per cell and the absorbed dose to the blood up to 5 h after ^177^Lu administration has been established. In the first hours after ^177^Lu administration, the average number of RIF per cell closely followed our in vitro calibration curve, thereby enabling the use of the DNA focus damage assay as an in vivo dosimeter. At 24 h and 48 h after ^177^Lu administration the mean number of RIF decreased, in accordance with the progression of DNA repair and declining dose rates.

## Electronic supplementary material

ESM 1 (PDF 125 kb)
