# Supplementary material for: DNA damage in blood lymphocytes in patients after 177Lu peptide receptor radionuclide therapy
Source: Eur J Nucl Med Mol Imaging. 2015 Jun 6;42(11):1739–49. doi: 10.1007/s00259-015-3083-9 (PMC4554740; doi:10.1007/s00259-015-3083-9)
Supplement: Supplementary file 1 — (PDF 125 kb) [file 259_2015_3083_MOESM1_ESM.pdf]

Eur J Nucl Med Mol Imaging
Doi: 10.1007/s00259-015-3083-9
Supplementary Material

DNA Damage in Blood Lymphocytes in Patients after ^{177}Lu Peptide Receptor Radionuclide Therapy

Supplementary Material

Uta Eberlein[1] · Carina Novak[2] · Christina Bluemel[1] · Andreas Konrad Buck[1] · Rudolf Alexander Werner[1] · Harry Scherthan[°2] · Michael Lassmann[°1]

Detailed description of Material and Methods

Blood sample preparation for the DNA damage focus assay

From each blood sample 4 ml of blood were recovered into a CPT Vacutainer tube (Becton Dickinson (BD) GmbH, Germany). One non-exposed sample was used to determine the individual background focus rate. The white blood cells were separated by 20 minute density centrifugation at 1500 g according to manufacturer's instructions (BD). Finally, the leucocytes were recovered above the interphase and washed twice in phosphate buffered saline (PBS). Ice-cold 99.9 % ethanol was added to the cell suspension to result in final concentration of 70 % ethanol. The fixed white blood cells were stored at least for 24 hours at -20°C and shipped to the Bundeswehr Institute of Radiobiology in Munich, Germany, where they were subjected to two-color immunofluorescent staining for γ-H2AX and 53BP1 [1]. The average frequencies of the co-localizing γ-H2AX/-53BP1 foci/nucleus were counted manually for 100 cells per sample by an experienced observer (H.S.) using a Zeiss Axioimager 2i epifluorescence microscope equipped with a red/green double band pass filter (AHF Analysentechnik, Germany). The standard deviation of the foci per cell data was calculated from the experiments assuming a Poisson distribution. The standard deviation of the background foci includes the counting error as well, therefore each data point is considered with appropriate error propagation, even the baseline value of the RIF/cell at 0 mGy. The number of radiation-induced damage foci (RIF) per cell was then obtained by subtracting, for each sample, the background foci rate.

First and Corresponding author
Uta Eberlein
E-mail: eberlein_u@ukw.de

[1] Department of Nuclear Medicine, University of Würzburg, 97078 Würzburg Germany
[2] Bundeswehr Institute of Radiobiology affiliated to the University of Ulm, 80937 Munich, Germany
° ML and HS share senior authorship

Calculation of the time-integrated activity coefficients and the absorbed doses

The curves describing the activity in blood (bl) and total body (tb) as a function of time after the administration usually are multi-exponential. Most of the activity is rapidly excreted with an effective half-life of typically a few hours, but smaller fractions are retained in accumulating tissues (e.g. tumour, liver, spleen). Since for this study the absorbed doses were calculated for the first 48 h after administration, a bi-exponential fit-function was adequate to determine the function describing the activity as a function of time for total body and blood.

$$\mathrm{FIA}(t) = \frac{A(t)}{A_0} = \sum_{\mathrm{i}=1}^{2} A_{\mathrm{i}} e^{-\lambda_{\mathrm{i}} t} \quad \text{(S1)}$$

$\mathrm{FIA}(t)$: Fraction of the administered activity A_0 as a function of time t; A_i and $\lambda_\mathrm{i} = \lambda_\mathrm{phys} + \lambda_\mathrm{i\,biol}$, are fit constants ($\lambda_\mathrm{phys}$ = 4.34E-03 h^{-1}), while A_i (units: $A_\mathrm{bl\,i}$: ml^{-1}, $A_\mathrm{tb\,i}$: dimensionless) defines the initial fraction of activity and λ_i (units: h^{-1}) defines the decay rate including biological and physical decay.
The time-integrated activity coefficients for the total body and activity concentration in blood, $\tau_\mathrm{tb}(t)$ and $\tau_\mathrm{ml\,of\,bl}(t)$, are calculated by integrating the respective retention functions $\mathrm{FIA}(t) = \frac{A(t)}{A_0}$ from 0 to t:

$$\tau(t) = \int_0^t \mathrm{FIA}(t)dt = \sum_{\mathrm{i}} \frac{A_\mathrm{i}}{\lambda_\mathrm{i}}(1 - e^{-\lambda_\mathrm{i} t}) \quad \text{(S2)}$$

Examples for the time activity curves of the blood and the total body, and the corresponding bi-exponential fits for the

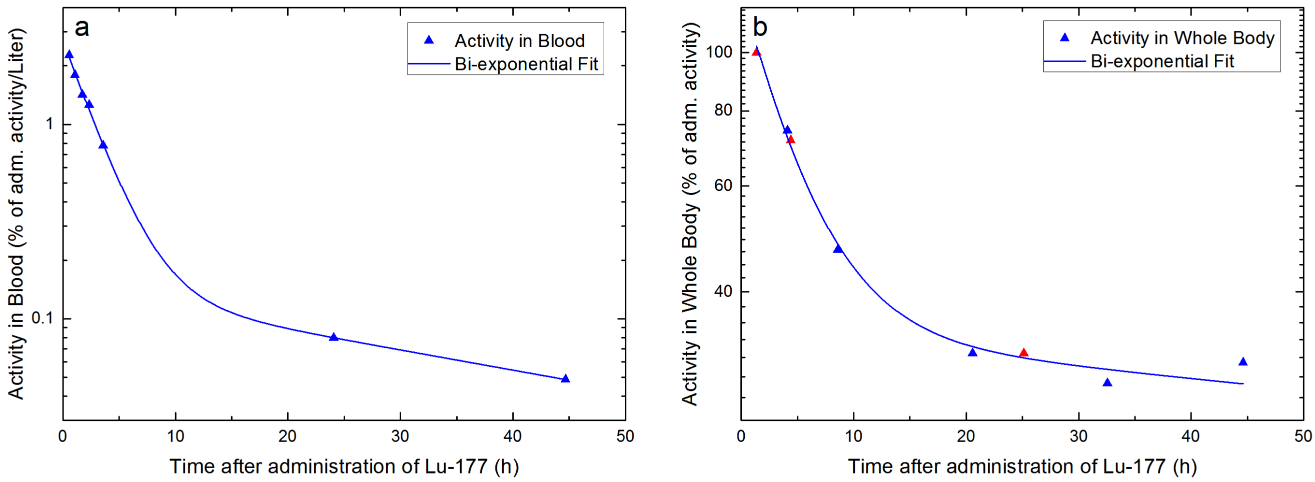

Fig. S1 Time activity curves for patient Lu3. a: time activity curve for the blood (% of administered activity/liter over time). b: time activity curve for the total body (% of administered activity over time). The blue triangles represent the dose-rate measurements and the red triangles represent the gamma camera measurements at three different time points.

patient Lu3 are shown in Figure S1. The fitting parameters of the individual patients are given in Table S1.

The absorbed doses were calculated in analogy to the EANM SOP for differentiated thyroid cancer (DTC) [2]. According to the generally accepted MIRD (Medical Internal Radiation Dose) [3] formalism, the mean absorbed dose to the blood $\overline{D}_{\mathrm{blood}}$ was determined by summing the contributions of blood self-irradiation and penetrating radiation from the total body multiplied by the administered activity (A_0):

$$\overline{D}_{\mathrm{bl}}(t) = A_0 \cdot (S_{\mathrm{bl}\leftarrow\mathrm{bl}} \cdot \tau_{\mathrm{ml\,of\,bl}}(t) + S_{\mathrm{bl}\leftarrow\gamma\,\mathrm{tb}} \cdot \tau_{\mathrm{tb}}(t)) \qquad \text{(S3)}$$

The S-value for the blood self-irradiation was calculated assuming that all β-energy is deposited in the blood neglecting γ-radiation. The maximum radiation absorption of the β-particles is 148 keV per decay corresponding to an absorbed dose of 85.3 Gy·ml/(GBq·h). This is in good agreement with the data published by Hänscheid et al. [4] who performed a Monte-Carlo-simulation of blood self-irradiation, resulting in a total absorbed dose of 87.8 Gy·ml/(GBq·h) in 1 ml of blood.

The S-value contribution for penetrating radiation from the total body was approximated by $S_{\mathrm{bl}\leftarrow\gamma\,\mathrm{tb}} \approx S_{\mathrm{tb}\leftarrow\gamma\,\mathrm{tb}}$ (note that only the γ-contribution was considered here) and calculated according to a method described in [2] by taking the weight-adapted γ-contribution to the total body S-value from OLINDA/EXM [5]:

$$S_{\mathrm{bl}\leftarrow\gamma\,\mathrm{tb}} = \frac{0.00185}{\mathrm{wt}^{2/3}} \frac{\mathrm{Gy}\cdot\mathrm{kg}^{2/3}}{\mathrm{GBq}\cdot\mathrm{h}} \qquad \text{(S4)}$$

wt denotes the patient's weight in kg.

This function assumes irradiation by homogeneous distribution of the activity in the body. If the activity is inhomogeneously distributed in the body, the S-value for penetrating radiation changes less than 15 %. Moreover, the gamma emission probability of ^{177}Lu is only about 17 %; therefore, the contribution of the second term in equation S3 to the total absorbed dose to the blood is low in the first 48 h after therapy.

References

1. Lamkowski A, Forcheron F, Agay D, et al. DNA Damage Focus Analysis in Blood Samples of Minipigs Reveals Acute Partial Body Irradiation. PLoS ONE 2014;9:e87458.
2. Lassmann M, Hänscheid H, Chiesa C, Hindorf C, Flux G, and Luster M. EANM Dosimetry Committee series on standard operational procedures for pre-therapeutic dosimetry I: blood and bone marrow dosimetry in differentiated thyroid cancer therapy. Eur J Nucl Med Mol Imaging 2008;35:1405–1412.
3. Bolch WE, Eckerman KF, Sgouros G, and Thomas SR. MIRD pamphlet No. 21: a generalized schema for radiopharmaceutical dosimetry–standardization of nomenclature. J Nucl Med 2009;50:477–484.
4. Hänscheid H, Fernández M, Eberlein U, and Lassmann M. Self-irradiation of the blood from selected nuclides in nuclear medicine. Phys Med Biol 2014;59:1515–1531.
5. Stabin MG, Sparks RB, and Crowe E. OLINDA/EXM: the second-generation personal computer software for internal dose assessment in nuclear medicine. J Nucl Med 2005;46:1023–1027.

Table S1 Fitting Parameters

			Blood	Blood	Blood	Blood	Total Body	Total Body	Total Body	Total Body
Pat-ID	λ* (h^{-1})	m*	A_{bl1}** (ml^{-1})	A_{bl2}** (ml^{-1})	λ_{bl1}** (h^{-1})	λ_{bl2}** (h^{-1})	A_{tb1}**	A_{tb2}**	λ_{tb1}** (h^{-1})	λ_{tb2}** (h^{-1})
Lu1	0.0553	1.86	1.10E-06	2.03E-05	3.19E-02	4.51E-01	9.07E-01	3.18E-01	2.99E-01	4.34E-03
Lu2	0.0295	1.14	2.02E-06	6.12E-05	3.27E-02	1.92E+00	2.92E-01	7.71E-01	1.56E-01	1.00E-02
Lu3	0.0313	0.83	1.41E-06	2.55E-05	2.38E-02	3.79E-01	9.18E-01	3.39E-01	2.08E-01	4.34E-03
Lu4	0.0407	2.84	2.02E-06	2.47E-05	3.53E-02	1.45E+00	8.83E-01	6.00E-01	4.93E-01	4.34E-03
Lu5	0.0506	0.55	2.28E-06	3.05E-05	1.33E-02	3.64E-01	3.82E-01	8.04E-01	3.02E-01	4.34E-03
Lu6	0.0673	1.95	2.05E-06	1.93E-05	1.93E-02	4.98E-01	5.08E-01	5.85E-01	3.97E-01	1.36E-02
Lu7	0.0345	0.82	1.37E-06	4.45E-05	2.43E-02	4.04E-01	1.07E+00	3.24E-02	2.50E-01	4.34E-03
Lu8	0.0424	1.91	1.69E-06	1.61E-06	1.39E-02	3.93E-01	2.67E-01	7.14E-01	1.72E-01	5.95E-03
Lu10	0.0261	1.54	2.12E-06	2.87E-05	2.41E-02	5.14E-01	6.11E-01	5.25E-01	3.40E-01	8.87E-03
Lu11	0.0287	0.94	1.59E-06	2.52E-05	3.01E-02	3.47E-01	8.47E-01	3.75E-01	3.39E-01	1.16E-02
Lu12	0.0366	1.23	1.99E-06	2.52E-05	4.61E-02	3.52E-01	1.09E+00	1.88E-01	3.06E-01	1.87E-02
Lu13	0.0234	0.60	1.50E-06	1.88E-05	2.35E-02	2.80E-01	5.85E-01	4.52E-01	2.35E-01	3.03E-02
Lu14	0.0140	0.63	9.39E-07	3.27E-05	2.33E-02	3.03E-01	1.00E+00	2.18E-01	2.88E-01	3.63E-02
Lu16	0.0194	0.56	1.28E-06	2.28E-05	3.14E-02	2.36E-01	1.18E+00	3.34E-01	4.12E-01	3.33E-02
Lu17	0.0218	1.22	1.52E-06	1.99E-05	4.24E-02	2.95E-01	9.49E-01	1.44E-01	2.07E-01	4.34E-03
Lu18	0.0841	1.81	3.51E-06	8.95E-06	1.73E-02	3.73E-01	4.32E-01	6.56E-01	3.08E-01	4.34E-03
Mean	0.0379	1.28	1.77E-06	2.56E-05	2.71E-02	5.35E-01	7.45E-01	4.41E-01	2.95E-01	1.25E-02
SD#	0.0187	0.66	6.06E-07	1.35E-05	9.38E-03	4.63E-01	2.86E-01	2.14E-01	9.06E-02	1.12E-02

* For an explanation of the symbols see equation 2 main article, ** see equations S1 and S2 , # SD= standard deviation.
